# Reference values for the cervical spinal canal and the vertebral bodies by MRI in a general population

**DOI:** 10.1371/journal.pone.0222682

**Published:** 2019-09-27

**Authors:** Christopher Nell, Robin Bülow, Norbert Hosten, Carsten Oliver Schmidt, Katrin Hegenscheid

**Affiliations:** 1 Department of Diagnostic Radiology and Neuroradiology, University Medicine Greifswald, Greifswald, Mecklenburg-Western Pomerania, Germany; 2 Institute for Community Medicine, University Medicine Greifswald, Greifswald, Mecklenburg-Western Pomerania, Germany; Rush University Medical Center, UNITED STATES

## Abstract

**Purpose:**

To provide population-based reference values for cervical spinal canal parameters and vertebral body (VB) width and to study their associations with sex, age, body height, body weight and body mass index (BMI) using MRI.

**Methods:**

Cross-sectional analyses included data from 2,453 participants, aged 21–89 years, of the population-based Study of Health in Pomerania (SHIP) who underwent whole-body MRI at 1.5 Tesla between July 2008 and March 2011. A standardised reading was performed for the C2-C7 cervical spine levels at sagittal T2 TSE weighted sequences.

**Results:**

Reference intervals for spinal canal parameters were similar in males and females, while VB width was on average 2.1–2.2 mm larger in males. Age effects were only substantial regarding VB width with a 0.5 mm per ten-year age increase. Body height effects were only substantial regarding the osseous spinal canal and VB width. Body weight and BMI effects are mostly not substantial.

**Conclusions:**

Our study provides MRI-based reference values for the cervical spinal canal parameters in an adult Caucasian population. Except for VB width, associations with sex, age and somatometric measures are mostly small and thus have only limited clinical implications. Some available cut-off values may need a revision because they likely overestimate risks.

## Introduction

MRI plays a crucial role in the assessment of cervical spine disorders [1–3]. Yet, reference values for the spinal canal derived from MRI are still not available. This complicates a proper understanding of what a normal morphological finding distinguishes from an abnormal one. Previous studies related to the cervical spinal canal are limited with regards to inferences about the general population due to: a) failure to report reference ranges based on adequate percentiles, i.e. the 2.5^th^ and 97.5^th^ percentiles [4–9]; b) small sample sizes [4, 6–9]; c) small age ranges [6–8]; and d) sampling in specific populations (e.g., only men or medical staff) [4, 6, 9]. There is a need for population-based data to better understand the distribution of spinal canal parameters in adult populations [4].

Few studies have investigated the role of additional predictors besides the spinal level such as sex, age, body height, body weight, and body mass index (BMI) [4, 5, 10–12]. Again, the study samples were not drawn from a general population and the findings were inconclusive. It remains unclear whether these parameters are associated with the spinal canal parameters.

Several parameters which might differentiate normal from aberrant spinal canals exist–such as the Torg Ratio (TR) [13, 14] or the space available for the cord (SAC) [6]. Therefore we provide population-based MRI reference values, defined as the interval that 95 percent of values of all healthy subjects fall into [15], for the cervical osseous spinal canal (OSC), dural sac (DS), spinal cord (SC), vertebral body (VB) width, TR, and SAC. Furthermore, we study the association of spinal canal parameters with spinal level, sex, age, body height, body weight, and BMI. In contrast to previous studies, we based our analyses on participants from a general population cohort.

## Materials and methods

### Study sample

Our cross-sectional analyses were based on the Study of Health in Pomerania (SHIP). SHIP was the first study to implement whole-body MRI (wb-MRI) in a general population sample [16]. All participants were drawn from regional population registries. SHIP comprises two independent cohorts: the second follow-up examination of the initial SHIP cohort (SHIP-2) and the baseline examination of an independent cohort established 2008 (SHIP-Trend-0). Both cohorts share the same inclusion criteria: age 20 to 79 years and primary place of residence in West Pomerania. Detailed response statistics are available elsewhere [17]. All participants gave written informed consent. The Medical Ethics committee of the University of Greifswald approved the study protocol. For more details, see [16, 18].

**Fig 1:**
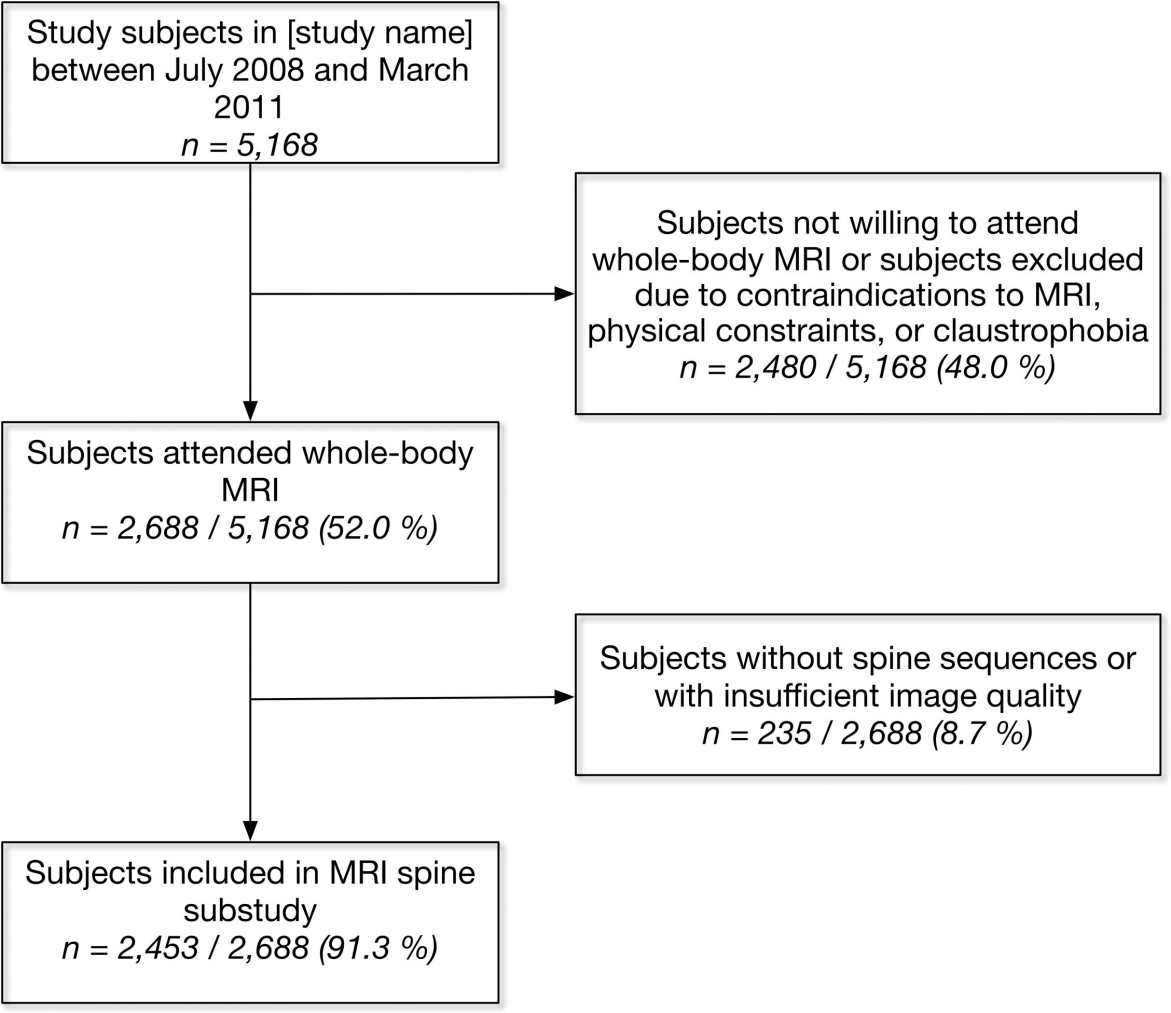
Study flow chart.

Between July 2008 and March 2011, 5,168 SHIP-2 and SHIP-Trend-0 participants were examined, of whom 2,688 (52.0%) underwent wb-MRI. 2,480 individuals were not willing to attend wb-MRI or were excluded due to contraindications to MRI (for example metallic implants), physical constraints, or claustrophobia. More information on the sample is depicted in Fig 1.

### MRI

Wb-MRI was performed according to a standardised protocol on a 1.5-Tesla MRI system (Magnetom Avanto, Siemens Healthcare, Erlangen, Germany), using integrated coil elements and a body phased-array coil [16]. Subjects were positioned supine with arms next to the body. The entire spine was imaged using a sagittal T2-weighted turbo spin echo (TSE) sequence (time-to-repetition 3,760 ms; time-to-echo 106 ms; flip angle 180°; field of view 500 x 500; matrix 448 x 448; slice thickness 4 mm; slice gap 0.4 mm; bandwidth 150 Hz/pixel) and a sagittal T1-weighted TSE sequence (time-to-repetition 676 ms; time-to-echo 12 ms; flip angle 150°; field of view 500 x 500; matrix 448 x 448; slice thickness 4 mm; slice gap 0.4 mm; bandwidth 150 Hz/pixel) in two stations.

### Image reading

First, the quality of all images was rated. Sufficient quality was defined as clear depiction of the boundaries of the OSC, DS, and SC without significant artefacts, while insufficient quality was defined as incomplete depiction of the boundaries of one of these structures or abortion of the MRI before completion of the spine sequences. Subjects with insufficient image quality (n = 235; 8.7%) were excluded from the analysis (Fig 1).

**Fig 2:**
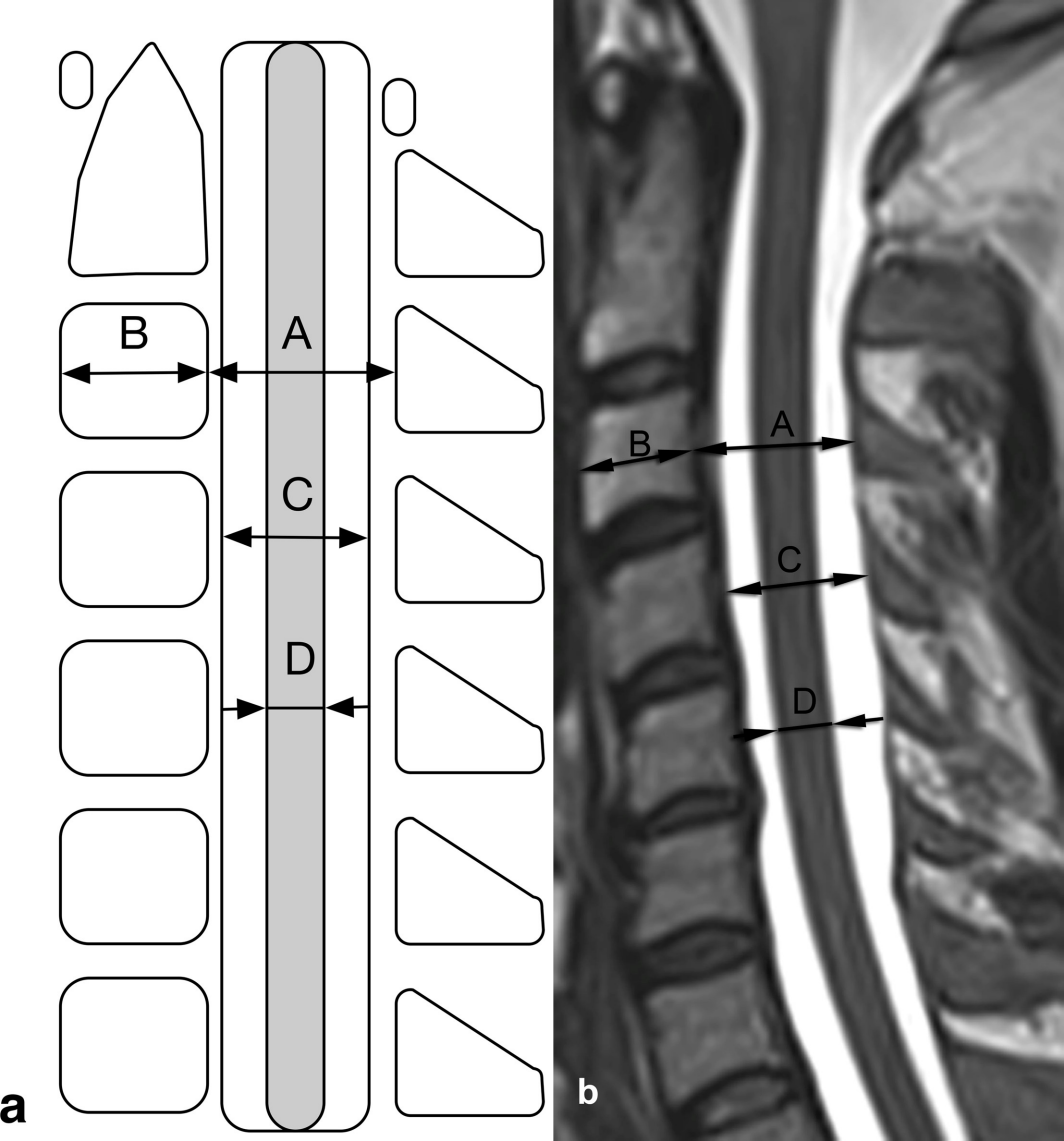
Measured items and differentiation between absolute and relative spinal stenosis. (a) Illustration. (b) Mid-sagittal T2-weighted turbo spin echo sequence. The anterior-posterior diameter of the osseous spinal canal was measured from the back of the craniocaudal center of each vertebral body to the nearest point on the corresponding spinolaminar line (line A) [20]. The anterior-posterior diameter of the dural sac was measured at the craniocaudal center of each vertebral body perpendicular to the dural sac (line C). To distinguish between dural sac and epidural fat, T1-weighted TSE sequences were used. The anterior-posterior diameter of the spinal cord was measured at the craniocaudal center of each vertebral body perpendicular to the spinal cord (line D). The anterior-posterior diameters of the vertebral bodies were measured between the anterior and posterior craniocaudal centers of the vertebral bodies (line B).

Second, standardised spinal canal measurements were performed midsagittal at the craniocaudal center of the vertebral body. This comprised the anterior-posterior diameters of the OSC, DS, SC and the VB at each vertebral level from C2 to C7 (Fig 2). The SAC was defined as the difference between the sagittal diameter of the DS and the sagittal diameter of the corresponding SC for each level from C2 to C7 [6]. The TR was defined as the ratio of the sagittal diameter of the OSC and the sagittal diameter of the corresponding vertebral body for each level from C2 to C7 [13, 14, 19].

All readings were performed by one resident radiologist (C.N.) with more than 6 years of experience using IMPACS ES 5.2 (AGFA Healthcare, Mortsel, Belgium) and were documented in a standardised web-based reading protocol. The reader was blinded to all other medical information. The MR images of a set of 50 randomly chosen individuals were measured twice, one month apart, and independently by the resident radiologist and a senior radiologist (K.H.) to assess intra- and inter-reader reliability. These results are reported in S1 and S2 Tables, respectively, and show good intra- and inter-reader reliability.

### Statistical analysis

To account for potential selection bias all reported results are based on inverse-probability-weighted data. Weights accounted for sex, age, occupational status, healthcare consultation, and subjective health status as measured with the first SF-12 [21] item and the SF-12 mental und physical summary component summary score.

The 2.5^th^ percentile and the 97.5^th^ percentile of the measured values defined reference intervals.

Estimates and confidence intervals for age, sex, body weight and body height effects were derived from weighted linear regression models using robust standard errors. Age effects were checked for nonlinearities using fractional polynomials, allowing for the powers (-2–1(.5)1 2 3) and up to 4 degrees of freedom [22]. Decisions between models were made based on a p-value of .01. A post-hoc analysis for the achieved power revealed that in a sample size of 2,453 even very small effect sizes (computed as Cohen's f^2^ = .01) will be detected at an alpha = .05 with a power >.99. Interpretations of results should rely primarily on the assumed clinical importance of the observed effect sizes instead of their statistical significance.

The imaging data were complete for all 2,453 included MRI participants.

P-values < .05 were described as being statistically significant. Statistical analyses were performed with STATA 14 (2016; StataCorp LP, College Station, Texas, U.S.).

## Results

### Study sample

There were no clinically relevant differences between MRI participants and non-MRI participants regarding demographic and somatometric measures (Table 1, Fig 1).

**Table 1:** Characteristics of the study sample by MRI participation.

Characteristics	MRI N = 2,453	Non-MRI N = 2,480
Sex (males / females)	1,194 / 1,259 (48.7 % / 51.3 %)	1,147 / 1,333 (46.25 % / 53.75 %)
Age (years)	53.2 (13.8)	54.9 (16.3)
body height (cm)	169.9 (9.3)	168.8 (9.3)
body weight (kg)	80.3 (15.1)	81.3 (17.0)
BMI (kg/m^2^)	27.8 (4.4)	28.5 (5.4)
Waist circumference (cm)	89.9 (12.9)	92.1 (15.0)
Hip circumference (cm)	101.9 (9.6)	103.5 (11.1)

Data are given as absolute number (percentage) or mean (standard deviation).

BMI = body mass index; MRI = magnetic resonance imaging.

Mean age, body height, body weight and BMI in the study sample and in both sexes are shown in Table 2.

**Table 2:** Mean age, body height, body weight and BMI in the study sample and in both sexes.

Characteristics	both sexes	Male	Female
	N = 2,453	N = 1,194	N = 1,259
Age (years)	53.2	53.4 (21–89)	53 (21–83)
body height (cm)	169.9	177 (156–197)	164 (139–189)
body weight (kg)	80.3	87.8 (53.3–142.7)	73.2 (41.5–126.1)
BMI (kg/m^2^)	27.8	28.15 (17.74–41.96)	27.38 (17.25–48.05)

Data are given as mean with ranges in parentheses.

BMI = body mass index; MRI = magnetic resonance imaging.

### Reference interval for spinal canal parameters and vertebral body width

Reference interval boundaries for OSC ranged from 12 to 21 mm in males (min 10 – max 23 mm) and from 12 to 20 mm in females (min 10 –max 23 mm), for DS from 10 to 17 mm in males (min 7 – max 19 mm) and from 9 to 16 mm in females (min 7 –max 18 mm), for SC from 5 to 9 mm in males (min 4 – max 10 mm) and from 5 to 9 mm in females (min 5 –max 11 mm), for VB from 14 to 22 mm in males (min 12 – max 27 mm) and from 12 to 19 mm in females (min 10 –max 25 mm), for SAC from 3 to 10 mm in males (min 0 – max 13 mm) and from 2 to 9 mm in females (min 0 –max 11 mm), and for TR from 0.60 to 1.36 in males (min 0.45 – max 1.83) and from 0.67 to 1.58 in females (min 0.55 –max 1.92) (Table 3).

**Table 3:** Sex-specific percentiles (P) of the osseous spinal canal (OSC), the dural sac (DS), the spinal cord (SC), the vertebral body (VB), the space available for the cord (SAC), and the Torg ratio (TR).

	Male (N = 1,194)									Female (N = 1,259)								
Items	P 0	P 2.5	P 5	P 25	P 50	P 75	P 95	P 97.5	P 100	P 0	P 2.5	P 05	P 25	P 50	P 75	P 95	P 97.5	P 100
**OSC (mm)**																		
C2	11	15	16	17	18	19	20	21	23	12	15	15	17	18	18	20	20	23
C3	11	13	13	15	16	17	18	19	22	12	13	13	15	16	17	18	18	21
C4	10	12	13	15	16	17	18	18	22	10	13	13	14	15	16	18	18	20
C5	11	12	13	14	15	16	18	18	21	11	12	13	14	15	16	17	18	23
C6	10	12	13	14	15	16	18	19	21	10	12	13	14	15	16	17	17	20
C7	12	13	14	15	16	17	18	19	22	11	13	13	14	15	16	17	18	19
**DS (mm)**	** **									** **								
C2	10	11	11	12	13	15	16	17	18	9	11	11	12	13	15	16	16	18
C3	8	10	11	12	13	14	16	16	19	9	10	11	12	13	13	15	16	18
C4	7	10	10	12	13	14	16	16	19	9	10	10	11	13	13	15	16	17
C5	8	10	10	11	13	14	15	16	19	8	9	10	11	12	13	15	15	17
C6	8	10	10	12	13	14	15	16	18	7	9	10	11	12	13	15	15	18
C7	10	11	11	13	14	15	16	17	19	9	11	11	12	13	14	15	16	18
**SC (mm)**	** **									** **								
C2	5	6	7	7	8	8	9	9	10	6	6	7	7	8	8	9	9	10
C3	5	6	6	7	7	8	9	9	9	6	6	6	7	7	8	8	9	9
C4	5	6	6	7	7	8	8	9	10	5	6	6	7	7	8	8	9	10
C5	5	6	6	7	7	8	8	9	9	5	6	6	7	7	8	8	8	11
C6	5	6	6	6	7	7	8	8	9	5	6	6	6	7	7	8	8	10
C7	4	5	6	6	7	7	8	8	9	5	5	6	6	7	7	8	8	9
**VB (mm)**	** **	** **	** **	** **	** **	** **	** **	** **	** **	** **	** **	** **	** **	** **	** **	** **	** **	** **
C2	12	15	15	16	17	18	19	20	23	11	12	13	14	15	16	17	17	19
C3	12	14	15	16	17	18	19	20	26	11	13	13	14	15	16	17	17	19
C4	12	14	15	16	17	18	20	21	26	10	13	13	14	15	16	17	18	20
C5	12	14	14	16	17	18	20	22	25	10	13	13	14	15	16	18	18	21
C6	12	14	15	16	18	19	21	22	27	11	13	13	14	15	16	18	19	25
C7	13	15	15	17	18	19	20	21	25	12	13	13	15	15	16	18	18	21
**SAC (mm)**	** **									** **								
C2	2	3	4	5	6	7	9	10	11	2	3	3	5	6	7	9	9	11
C3	1	3	3	4	5	7	8	9	11	2	3	3	4	5	6	8	9	11
C4	0	3	3	4	5	7	8	9	11	1	3	3	4	5	6	8	9	10
C5	1	3	3	4	5	7	8	9	11	0	2	3	4	5	6	8	8	10
C6	0	3	3	5	6	7	8	9	11	0	2	3	4	5	6	8	8	11
C7	3	4	5	6	7	8	10	10	13	2	4	4	5	6	7	9	9	11
**TR **	** **									** **								
C2	0.58	0.83	0.85	1	1.06	1.17	1.31	1.36	1.83	0.72	0.94	0.94	1.07	1.2	1.29	1.5	1.58	1.92
C3	0.5	0.68	0.72	0.88	0.94	1.06	1.2	1.25	1.58	0.67	0.76	0.82	1	1.07	1.2	1.38	1.38	1.64
C4	0.48	0.65	0.68	0.83	0.93	1	1.14	1.21	1.58	0.63	0.75	0.78	0.94	1	1.14	1.31	1.38	1.6
C5	0.46	0.62	0.67	0.81	0.89	1	1.14	1.21	1.42	0.57	0.7	0.74	0.88	1	1.13	1.29	1.33	1.77
C6	0.45	0.6	0.65	0.76	0.88	1	1.13	1.21	1.5	0.55	0.67	0.71	0.87	0.94	1.07	1.23	1.29	1.54
C7	0.5	0.68	0.71	0.83	0.89	1	1.13	1.2	1.4	0.62	0.72	0.78	0.88	1	1.07	1.21	1.29	1.46

### Associations of spinal canal parameters and vertebral body width with sex and age

**Fig 3:**
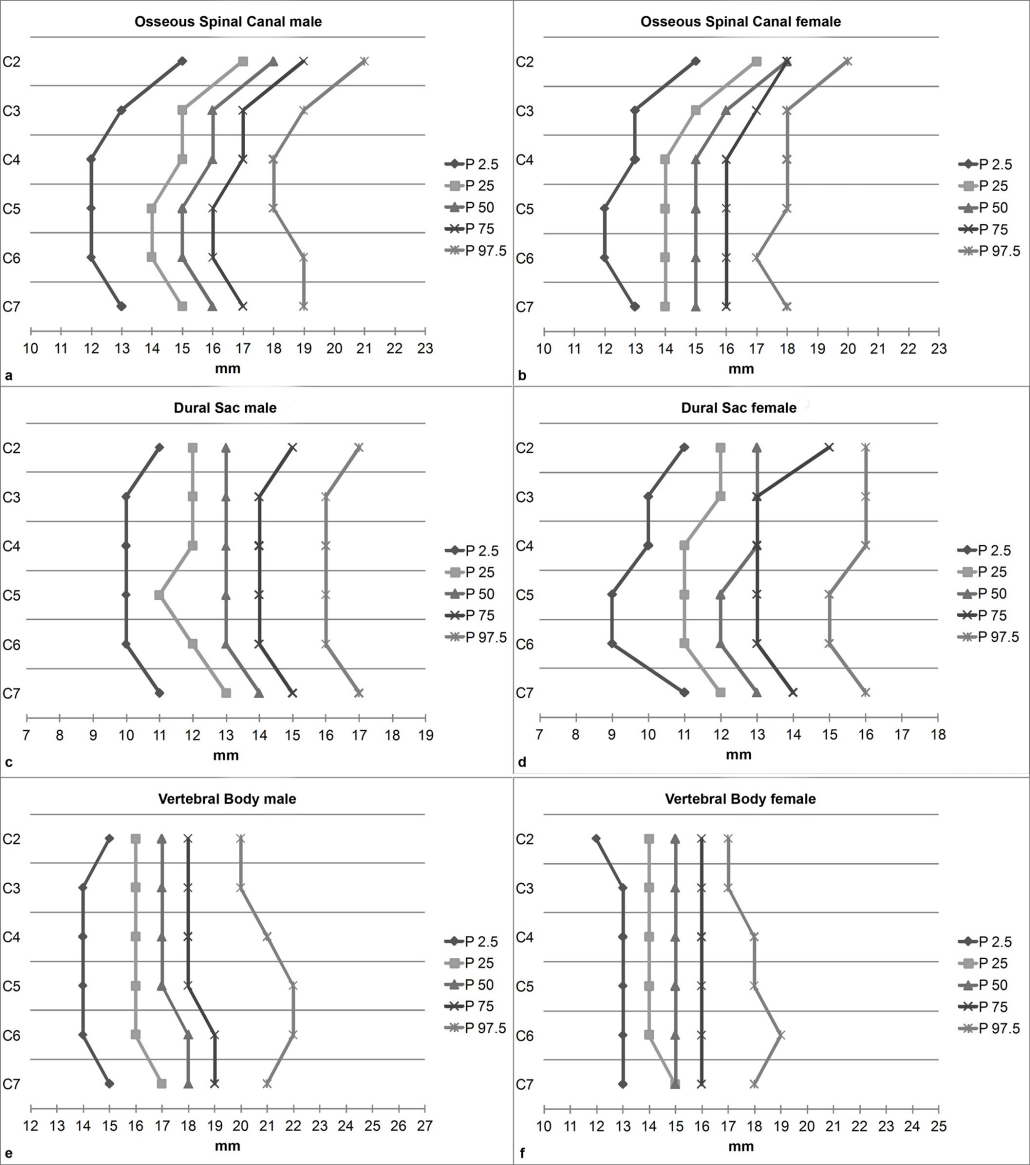
**Sex-specific reference values for the osseous spinal canal (a, b), the dural sac (c, d) and the vertebral bodies (e, f).** P denotes percentiles.

**Fig 4:**
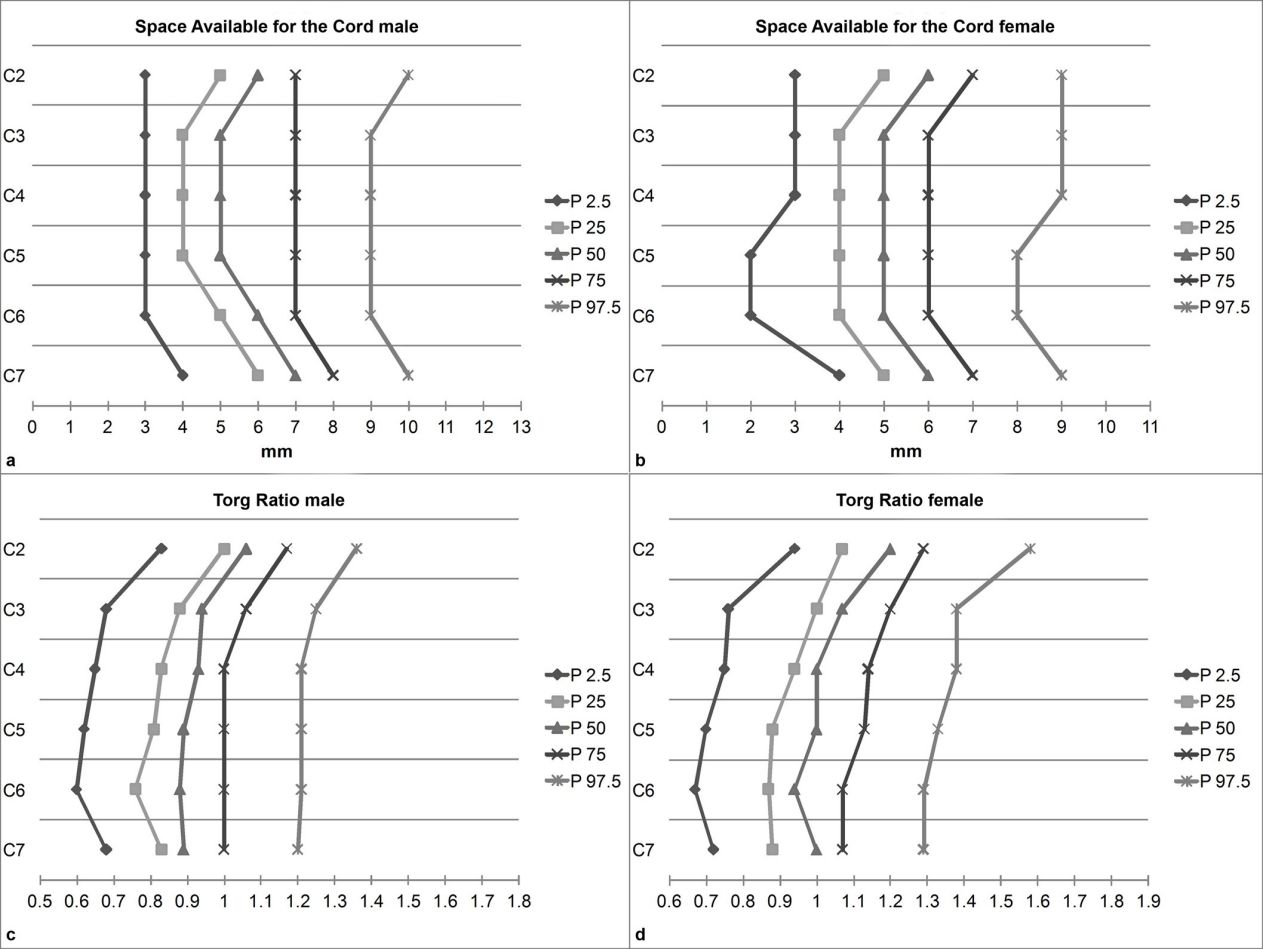
**Sex-specific reference values for the space available for the cord (SAC) (a, b) and the Torg ratio (TR) (c, d).** P denotes percentiles.

Measurements for SC were nearly identical for both sexes at all studied percentiles (Table 3, Fig 3). For OSC, DS, and SAC, the measurements were very similar between both sexes as well (Table 3, Figs 3 and 4). Substantial sex differences were found for VB width, with males having on average 2.1–2.2 mm (95% confidence intervals (CI) ranging from 1.94 to 2.30; p < 0.001) larger VBs. Related effect sizes and CIs are presented in Table 4. Torg ratios were consistently lower in males compared to females by 0.07–0.13 (CIs ranging from 0.06 to 0.14; p < 0.001).

Age has mostly statistically significant but clinically minor associations (effect sizes < 0.2 mm per ten-year age increase) with OSC, DS, SC, TR, and SAC measures. One exception is VB width, for which we found an association with age effects of up to 0.5 mm per ten-year age increase (CIs ranging from 0.13 to 0.56; p < 0.001) (Table 4). A check for nonlinear associations with age revealed only some nonlinear associations with SC but changes were below 1 mm across the entire age range and are therefore not reported.

**Table 4:** Effects of age (per 10 year age increase) and sex on the osseous spinal canal (OSC), the dural sac (DS), the spinal cord (SC), the vertebral body (VB), the space available for the cord (SAC), and the Torg ratio (TR).

Items	Age per 10 years Effect sizes^a^ and 95 % confidence intervals	Sex Effect sizes^a^ and 95 % confidence intervals
**OSC**		
C2	-0.19 (-0.24; -0.15)**	-0.34 (-0.46; -0.22)**
C3	-0.21 (-0.26; -0.17)**	-0.22 (-0.34; -0.10)**
C4	-0.21 (-0.26; -0.17)**	-0.36 (-0.48; -0.24)**
C5	-0.23 (-0.28; -0.19)**	-0.57 (-0.69; -0.45)**
C6	-0.21 (-0.25; -0.16)**	-0.72 (-0.84; -0.59)**
C7	-0.09 (-0.13; -0.05)**	-0.77 (-0.88; -0.66)**
**DS**		
C2	-0.20 (-0.25; -0.16)**	-0.37 (-0.51; -0.24)**
C3	-0.20 (-0.25; -0.15)**	-0.22 (-0.35; -0.10)**
C4	-0.20 (-0.25; -0.16)**	-0.16 (-0.29; -0.04)*
C5	-0.24 (-0.29; -0.20)**	-0.42 (-0.55; -0.30)**
C6	-0.24 (-0.29; -0.20)**	-0.46 (-0.59; -0.33)**
C7	-0.04 (-0.08; -0.00)*	-0.65 (-0.76; -0.54)**
**SC**		
C2	-0.04 (-0.07; -0.02)**	-0.01 (-0.06; 0.05)
C3	-0.07 (-0.09; -0.05)**	-0.01 (-0.07; 0.04)
C4	-0.10 (-0.12; -0.08)**	-0.05 (-0.10; 0.01)
C5	-0.11 (-0.13; -0.10)**	-0.10 (-0.16; -0.05)**
C6	-0.08 (-0.10; -0.06)**	-0.02 (-0.08; 0.04)
C7	-0.05 (-0.08; -0.03)**	0.00 (-0.06; 0.06)
**VB**		
C2	0.17 (0.13; 0.21)**	-2.11 (-2.22; -2.01)**
C3	0.22 (0.18; 0.26)**	-2.05 (-2.16; -1.94)**
C4	0.32 (0.28; 0.36)**	-2.06 (-2.17; -1.95)**
C5	0.48 (0.44; 0.53)**	-2.17 (-2.30; -2.04)**
C6	0.50 (0.45; 0.56)**	-2.16 (-2.30; -2.02)**
C7	0.33 (0.29; 0.37)**	-2.08 (-2.21; -1.96)**
**SAC**		
C2	-0.16 (-0.21; -0.11)**	-0.36 (-0.50; -0.23)**
C3	-0.13 (-0.18; -0.09)**	-0.22 (-0.34; -0.09)**
C4	-0.10 (-0.15; -0.06)**	-0.11 (-0.24; -0.01)
C5	-0.13 (-0.18; -0.09)**	-0.32 (-0.45; -0.19)**
C6	-0.16 (-0.21; -0.11)**	-0.44 (-0.57; -0.31)**
C7	0.01 (-0.03; 0.05)	-0.65 (-0.77; -0.53)**
**TR**		
C2	-0.02 (-0.03; -0.02)**	0.13 (0.12; 0.14)**
C3	-0.03 (-0.03; -0.02)**	0.12 (0.11; 0.13)**
C4	-0.03 (-0.04; -0.03)**	0.10 (0.09; 0.12)**
C5	-0.04 (-0.05; -0.04)**	0.09 (0.08; 0.10)**
C6	-0.04 (-0.04; -0.04)**	0.07 (0.06; 0.08)**
C7	-0.02 (-0.03; -0.02)**	0.07 (0.06; 0.08)**

We display unstandardised beta coefficients. Confidence intervals are given in parentheses.

^a^Effect sizes are provided in mm for all parameters except TR, which is dimensionless.

* p < 0.05

** p < 0.001.

### Associations of spinal canal parameters and vertebral body width with body height, body weight and BMI

Neither body height and body weight nor BMI affect the measurements of the DS and SC in a relevant manner (Table 5). The OSC is only affected by the body height with an increase of approx. 0.2 mm per 10-cm body height increase. Effects of body weight and BMI are negligible. The VB measurements are significantly associated with body height and body weight while BMI has no relevant effect size. Per 10-cm body height increase the VB measurement increases up to 0.81 mm (CIs ranging from 4.42 to 0.92 mm; p < 0.001).

**Table 5:** Effects of body height (per 10 cm increase), body weight (per 10 kg increase) and body mass index (BMI; per 1 kg/m^2^ increase) on the osseous spinal canal (OSC), the dural sac (DS), the spinal cord (SC), the vertebral body (VB), the space available for the cord (SAC), and the Torg ratio (TR).

Items	Body height effect sizes^a^ and 95% confidence intervals	Body weight effect sizes^a^ and 95% confidence intervals	BMI effect sizes^a^ and 95% confidence intervals
**OSC**			
C2	0.23 (0.13; 0.32)**	0.04 (-0.00; 0.09)	-0.00 (-0.01; 0.01)
C3	0.14 (0.05; 0.25)*	0.03 (*0.02; 0.07)	-0.00 (-0.01; 0.01)
C4	0.18 (0.08; 0.28)**	0.05 (-0.00; 0.09)	0.00 (-0.01; 0.02)
C5	0.13 (0.03; 0.23)*	0.06 (0.01; 0.11)*	0.01 (-0.00; 0.02)
C6	0.19 (0.09; 0.29)**	0.07 (0.02; 0.11)*	0.01 (-0.00; 0.02)
C7	0.28 (0.19; 0.38)**	0.04 (-0.00; 0.08)	-0.01 (-0.02; 0.01)
**DS**			
C2	0.05 (-0.06; 0.16)	-0.02 (-0.07; 0.03)	-0.01 (-0.03; 0.01)
C3	0.12 (0.01; 0.22)*	-0.00 (-0.47: 0.47)	-0.01 (-0.02; 0.01)
C4	0.13 (0.02; 0.23)*	0.03 (-0.02; 0.08)	0.00 (-0.01; 0.01)
C5	0.11 (-0.00; 0.21)	0.04 (-0.01;0.09)	0.01 (-0.01; 0.02)
C6	0.14 (0.03; 0.24)*	0.04 (-0.01; 0.09)	0.00 (-0.01; 0.02)
C7	0.21 (0.11; 0.30)**	0.02 (-0.02; 0.06)	-0.01 (-0.02; 0.01)
**SC**			
C2	-0.04 (-0.08; 0.01)	0.01 (-0.02; 0.03)	0.00 (-0.00; 0.01)
C3	-0.04 (-0.08; 0.01)	-0.00 (-0.02; 0.02)	0.00 (-0.01; 0.01)
C4	-0.04 (-0.08; 0.01)	-0.02 (-0.04; 0.00)	-0.00 (-0.01; 0.00)
C5	-0.06 (-0.10; -0.01)*	-0.02 (-0.01; 0.00)	-0.00(-0.01; 0.00)
C6	-0.02 (-0.07; 0.03)	-0.02 (-0.04; 0.00)	-0.01 (-0.01; 0.00)
C7	-0.03 (0.07; 0.02)	-0.04 (-0.06; -0.01)**	-0.01 (-0.02; -0.00)*
**VB**			
C2	0.51 (0.42; 0.60)**	0.13 (0.09; 0.17)**	0.01 (-0.00; 0.02)
C3	0.54 (0.46; 0.62)**	0.14 (0.11; 0.18)**	0.01 (-0.00; 0.02)
C4	0.57 (0.49; 0.66)**	0.15 (0.11; 0.19)**	0.01 (-0.00; 0.02)
C5	0.72 (0.62; 0.83)**	0.21 (0.16; 0.26)**	0.02 (0.00; 0.03)*
C6	0.81 (0.70; 0.92)**	0.25 (0.20; 0.30)**	0.03 (0.01; 0.04)**
C7	0.76 (0.66; 0.86)**	0.23 (0.18; 0.27)**	0.02 (0.01; 0.04)*
**SAC**			
C2	0.09 (-0.03; 0.20)	-0.03 (-0.08; 0.02)	-0.01 (-0.03; 0.00)
C3	0.15 (0.05; 0.26)*	0.00 (-0.04; 0.05)	-0.01 (-0.02; 0.00)
C4	0.16 (0.06;0.27)*	0.05 (-0.00; 0.01)	0.00 (-0.01; 0.02)
C5	0.16 (0.06; 0.27)*	0.06 (0.01; 0.11)*	0.01 (-0.01: 0.02)
C6	0.15 (0.05; 0.26)*	0.06 (0.01; 0.11)*	0.01 (-0.01; 0.02)
C7	0.23 (0.13; 0.34)**	0.06 (0.01; 0.10)*	0.00 (-0.01; 0.02)
**TR**			
C2	-0.02 (-0.03; -0.13)**	-0.01 (-0.01; -0.00)*	-0.00(-0.00; -0.00)
C3	-0.03 (-0.04; -0.02)**	-0.01 (-0.01; -0.00)**	-0.00(-0.00; 0.00)
C4	-0.02 (-0.03; -0.02)**	-0.01 (-0.01; -0.00)*	-0.00(-0.00; 0.00)
C5	-0.03 (-0.04; -0.02)**	-0.01 (-0.01; -0.00)**	-0.00(-0.00; 0.00)
C6	-0.03 (-0.04; -0.02)**	-0.01 (-0.01; -0.01)**	-0.00(-0.00; 0.00)
C7	-0.03 (-0.04; -0.02)**	-0.01 (-0.01; -0.01)**	-0.00(-0.00; -0.00)*

We display unstandardised beta coefficients (unit is mm except for TR). Confidence intervals are given in parentheses.

^a^Effect sizes are provided in mm for all parameters except TR, which is dimensionless.

* p < 0.05

** p < 0.001.

The SAC increases slightly in larger and heavier subjects. TR shows the opposite associations. BMI shows near-to-no effect on both SAC and TR (Table 5).

## Discussion

Our study advances the knowledge about the morphology of the cervical spinal canal and its demographic variability in two aspects. First, we are the first to provide population-based reference values for a wide range of spinal canal parameters and vertebral bodies of the entire cervical spine using MRI for an adult Caucasian population. Second, sex, age and somatometric measures show mostly minor associations to spinal canal parameters, except for VB.

OSC, DS and TR measures are smallest at the C6 level in terms of means and 2.5^th^ percentiles in both sexes. Correspondingly, C6 has the largest VB in both sexes regarding means and 97.5^th^ percentiles. This potentially puts lower cervical levels, particularly C6, at higher risk for developing symptomatic stenosis, as proposed previously [2, 3, 10, 12, 23, 24]. The SC dimension is nearly constant across all levels in both sexes. This may be attributable to the fact that the SC is part of the central nervous system and does not contribute to spinal stability. VBs in males are more than 2 mm larger than those in females across all levels. This and the significantly smaller TR could explain why males have a higher risk of myelopathy than females [2, 12, 23].

SAC size is assumed to indicate the functional reserve for SC movement or minor spinal canal changes due to aging, trauma or inflammation [6, 23, 24]. Interestingly, the levels with the smallest mean SAC are C4 in males and C5 in females, while we expected C6 to be the level with the smallest mean SAC. Nonetheless, the levels with the smallest 2.5^th^ percentiles regarding the SAC comprise C5 and C6 in both sexes as well. These values go down to 2 and 3 mm, respectively, and convey different information compared to the previous reported means of about 4 to 7 mm [4–6, 11]. This leads to two possible consequences: a) methodologically, for proper judgment about (normal) measurements one should use the reference range instead of mean values and b) clinically, it is doubtful that SAC is a good indicator for spinal stenosis.

Normal TR is supposed to be around 1.00, and ratios below 0.82 [13, 14] are usually interpreted to indicate stenosis. In our population, the median for both sexes is around 1.00 but the 2.5^th^ percentiles go down to 0.6 (C6) in males and to 0.67 (C6) in females. This suggests that available cut-off values overestimate the probability of having spinal stenosis and should be adapted accordingly [23, 25].

There is no widely-accepted definition of “normal” measurements for the cervical spinal canal measurements [4, 5]. To the best of our knowledge, previous studies based on MRI did not provide proper reference values. Most studies refer to mean values, but this does not describe the range of what might be considered “normal”. Furthermore, some previous values were transposed from radiographic studies to MRI without adaptation. An often cited definition is 13 mm for relative and 10 mm for absolute spinal stenosis [4, 7, 26, 27]. These thresholds are within the normal range or even below the lower reference range in our study. We suggest using the 2.5^th^ percentile for each level for the OSC, DS, SC, SAC and TR measurements; and the 97.5^th^ percentile for the VB measurements, respectively, as shown in Table 3. Yet the clinical importance of values outside the reference ranges require further clinical evaluation.

Ancestry is an additional factor which contributes to the size of the spinal canal and therefore must be take into account [28]. Compared with the results from a Japanese sample [5], which reported only means and standard deviations, our values for the OSC, DS, SC, and SAC are consistently higher. This may be due to Caucasian having larger body builds than Japanese and greater body height is associated with a wider spinal canal [4, 10, 29].

Previous investigations studied the correlation between age and spinal canal size with different results [4, 5, 10]. We show that sex and age have consistent associations with most spinal canal parameters and VB width. Effects are small in most cases and thus have only limited clinical implications. For example, the OSC dimension decreases approx. 1 mm across the entire age range for C2-C7, while the OSC range between the 2.5^th^ and 97.5^th^ percentiles is 9 mm. Only VB width and the VB-dependent TR are related to sex and age in a clinically relevant manner. Again, C6 shows the highest variability. The mean increases from 15.4 mm (at age of 30) to 18 mm (at age of 80). We therefore recommend taking patient age into account when assessing VB width and using the reference values identified here. Our reference values apply to the mean age in our sample. For each 10-year deviation in age from the mean, we suggest adapting the values for VB width with the age-related effect sizes provided in Table 4 using the formula: age effect*(patient age-50).

Body height effect sizes regarding the OSC were similar to age effect sizes. The effect sizes regarding VBs are even larger. Body height does not affect SC. The SAC is larger in larger subjects, this confirms findings of previous studies and therefore, smaller subjects tend to have a greater risk to develop symptoms due to even minor changes to the spinal canal [4, 10, 29]. TR is decreasing with body height, which is contrary to the findings regarding the SAC. Most likely the changes of the VB with the body height causes this decrease. The role of the TR to evaluate the spinal canal is controversial [4, 7, 25, 30, 31]. The diagnostic power of the TR is likely to be low, because this parameter relies heavily on the VB width.

The effect sizes of the body weight and BMI on spinal canal measures are mostly clinically irrelevant. This is somehow surprising because we expected a high BMI as a factor of spinal lipomatosis and therefore contributing to narrowing of the DS as it is shown for the lumbar spine recently [32]. But obesity in terms of high BMI is not linked with a narrow cervical spinal canal according to our findings.

Interindividual variation has more impact compared to the relatively small effect sizes of body height, body weight and BMI. For example, a moderate effect size of 0.19 mm increase of the OSC at C6 per 10-cm increase of the body height results in approx. 1 mm difference between the smallest and the tallest female whereas the difference between the 2.5^th^ and 97.5^th^ percentiles for the OSC at C6 for females is 5 mm. Adapting the measurements is not reasonable because of small effect sizes except for sex and partially age and body height.

This study used a commercially available 1.5 Tesla MRI system while currently 3 Tesla scanners are available for clinical routine use, too. A higher field strength may result on the one hand side in reduced examination times [33–35]. This is of little relevance in the context of this study. Furthermore, spatial resolution may be increased [33, 36]. Yet, any slightly increased resolution would not alter any key conclusions drawn in this study. Therefore we consider our results to be of relevance when using a 3 Tesla scanner.

Our study has several strengths and limitations. One strength is the large sample size drawn at random from a general population. This addresses the limitations of previous studies. Another strength is the good intra- and inter-reader reliability.

A limitation is the measurement of spinal parameters at the craniocaudal center of each vertebral body in the sagittal plane although most degenerative alterations of spinal canal dimensions might take place at the disc level. However, this is a standard procedure for studies examining the spine [4, 5, 7] because measurements at the disc level are less reliable [4, 6, 7] Furthermore, diameter measurements in sagittal planes are more reliable than measurements in axial planes [4]. This study did not intend to identify subjects at risk on an individual approach. Further studies should assess the potential of identifying patients at risk for spinal stenosis based on spinal canal parameters in a prospective case-control study or longitudinal setting.

In conclusion, our study provides MRI-based reference values for the cervical spinal canal parameters in an adult Caucasian population. Except for VB width associations with sex, age and somatometric measures are mostly small and thus have only limited clinical implications. Some available cut-off values may need a revision because they likely overestimate risks and might not be suitable to identify patients at risk of developing symptomatic spinal stenosis.

## Supporting information

S1 Table Intra-reader-reliability for spinal canal and vertebral body measurements for the two readers for a set of 50 randomly chosen individuals. Mean bias denotes the standardised mean difference between both readings for each reader in percent. 1.96*SD denotes the 1.96-fold standard deviation of the differences between both readings for each reader in percent. Limits of agreement were defined as mean bias <5% and 1.96*SD <25%. SD = standard deviation; OSC = osseous spinal canal; DS = dural sac; SC = spinal cord; VB = vertebral body.
(DOCX)

S2 Table Inter-reader-reliability for spinal canal and vertebral body measurements. Mean bias denotes the standardised mean difference between both readers in percent. 1.96*SD denotes the 1.96-fold standard deviation of the differences between both readers in percent. Limits of agreement were defined as mean bias < 5% and 1.96*SD <25%. SD = standard deviation; OSC = osseous spinal canal; DS = dural sac; SC = spinal cord; VB = vertebral body.
(DOCX)

## Abbreviations

BMI: body mass index
C1 …C7: cervical spine levels
CI: confidence interval
DS: dural sac
MRI: magnetic resonance imaging
OSC: osseous spinal canal
SAC: space available for the cord
SC: spinal cord
SHIP: Study of Health in Pomerania
TR: Torg ratio
VB: vertebral body
wb-MRI: whole body magnetic resonance imaging
