# Supplementary material for: Reference values for the cervical spinal canal and the vertebral bodies by MRI in a general population
Source: PLoS One. 2019 Sep 27;14(9):e0222682. doi: 10.1371/journal.pone.0222682 (PMC6764695; doi:10.1371/journal.pone.0222682)
Supplement: S1 Table — Mean bias denotes the standardised mean difference between both readings for each reader in percent. 1.96*SD denotes the 1.96-fold standard deviation of the differences between both readings for each reader in percent. Limits of agreement were defined as mean bias <5% and 1.96*SD <25%. SD = standard deviation; OSC = osseous spinal canal; DS = dural sac; SC = spinal cord; VB = vertebral body. (DOCX) [file pone.0222682.s001.docx]

S1 Table. Intra-reader-reliability for spinal canal and vertebral body measurements for the two readers for a set of 50 randomly chosen individuals

|  | **1^st^ reader** | |  | **2^nd^ reader** | |
| --- | --- | --- | --- | --- | --- |
| **Item** | **Mean bias (%)** | **1.96*SD (%)** |  | **Mean bias (%)** | **1.96*SD (%)** |
| **OSC** |  |  |  |  |  |
| C2 | 0.34 | 9.29 |  | -0.34 | 3.48 |
| C3 | 0.84 | 13.43 |  | -0.12 | 1.68 |
| C4 | 1.33 | 13.04 |  | -0.26 | 2.5 |
| C5 | 4.06 | 13 |  | -0.12 | 1.68 |
| C6 | 3.13 | 13.05 |  | 0.25 | 3.46 |
| C7 | 0.57 | 10.53 |  | -0.01 | 4.81 |
| **DS** |  |  |  |  |  |
| C2 | -3.98 | 12.83 |  | -0.17 | 5.16 |
| C3 | -1.26 | 14.7 |  | -0.7 | 7.58 |
| C4 | -0.29 | 15.55 |  | -0.67 | 6.47 |
| C5 | 0.01 | 12.88 |  | 0.31 | 4.26 |
| C6 | 1.09 | 17.23 |  | 0.16 | 2.22 |
| C7 | -3.24 | 16.77 |  | 0 | 0 |
| **SC** |  |  |  |  |  |
| C2 | 3.79 | 19.19 |  | -0.57 | 7.92 |
| C3 | 3.7 | 17.42 |  | -0.78 | 6.15 |
| C4 | 3.7 | 15 |  | -1.38 | 8.27 |
| C5 | 3.31 | 18.68 |  | 0.1 | 6.31 |
| C6 | -4.32 | 19.08 |  | 0.31 | 4.26 |
| C7 | -3.15 | 17.63 |  | 0.57 | 7.92 |
| **VB** |  |  |  |  |  |
| C2 | 1.52 | 12.71 |  | -0.25 | 2.43 |
| C3 | 3.08 | 13.92 |  | 0.25 | 4.69 |
| C4 | 2.9 | 11.4 |  | -0.25 | 5.63 |
| C5 | 2.07 | 11.08 |  | -0.19 | 4.74 |
| C6 | 2.44 | 13.67 |  | -0.23 | 3.94 |
| C7 | 1.56 | 12.87 |  | 1.45 | 16.32 |

Mean bias denotes the standardised mean difference between both readings for each reader in percent. 1.96*SD denotes the 1.96-fold standard deviation of the differences between both readings for each reader in percent. Limits of agreement were defined as mean bias <5 % and 1.96*SD <25 %.

SD = standard deviation; OSC = osseous spinal canal; DS = dural sac; SC = spinal cord; VB = vertebral body.
