# Supplementary material for: Reference values for the cervical spinal canal and the vertebral bodies by MRI in a general population
Source: PLoS One. 2019 Sep 27;14(9):e0222682. doi: 10.1371/journal.pone.0222682 (PMC6764695; doi:10.1371/journal.pone.0222682)
Supplement: S2 Table — Mean bias denotes the standardised mean difference between both readers in percent. 1.96*SD denotes the 1.96-fold standard deviation of the differences between both readers in percent. Limits of agreement were defined as mean bias < 5% and 1.96*SD <25%. SD = standard deviation; OSC = osseous spinal canal; DS = dural sac; SC = spinal cord; VB = vertebral body. (DOCX) [file pone.0222682.s002.docx]

S2 Table. Inter-reader-reliability for spinal canal and vertebral body measurements

| **Item** | **Mean bias (%)** | **1.96*SD (%)** |
| --- | --- | --- |
| **OSC** |  |  |
| C2 | -0.51 | 9.35 |
| C3 | -0.59 | 12.89 |
| C4 | -0.59 | 11.09 |
| C5 | -2.45 | 12.18 |
| C6 | -1.75 | 15.19 |
| C7 | -1.16 | 11.22 |
| **DS** |  |  |
| C2 | 1.37 | 13.65 |
| C3 | -0.43 | 13.77 |
| C4 | -2.44 | 13.45 |
| C5 | -4.91 | 15.36 |
| C6 | -4.23 | 14.44 |
| C7 | -4.7 | 12.02 |
| **SC** |  |  |
| C2 | 0.35 | 17.51 |
| C3 | 1.13 | 15.55 |
| C4 | 0.91 | 15.55 |
| C5 | -0.72 | 13.49 |
| C6 | -5.57 | 16.4 |
| C7 | -4.3 | 20.68 |
| **VB** |  |  |
| C2 | -2.27 | 10.52 |
| C3 | -0.48 | 12.16 |
| C4 | -0.65 | 10.43 |
| C5 | -0.54 | 11.34 |
| C6 | -2.12 | 13.46 |
| C7 | -0.77 | 13.69 |

Mean bias denotes the standardised mean difference between both readers in percent. 1.96*SD denotes the 1.96-fold standard deviation of the differences between both readers in percent. Limits of agreement were defined as mean bias < 5 % and 1.96*SD <25 %.

SD = standard deviation; OSC = osseous spinal canal; DS = dural sac; SC = spinal cord; VB = vertebral body.
